# Sporadic lymphangioleiomyomatosis in young female patient: A case report and review of the literature

**DOI:** 10.1016/j.ijscr.2025.111913

**Published:** 2025-09-04

**Authors:** Addisu Assfaw Ayen, Dereje Desta Mihretu, Amsalu Molla Getahun, Belayneh Dessie Kassa, Fetene Bezabih, Abebe Shumet

**Affiliations:** a Department of Internal Medicine, Debre Tabor University, Debre Tabor, Ethiopia; b Department of Internal Medicine, Pulmonology and Critical Care Unit, Bahir Dar University, Bahir Dar, Ethiopia; c Department of Surgery, Debre Tabor University, Debre Tabor, Ethiopia; d Department of Emergency and Critical Care Medicine, Debre Tabor University, Debre Tabor, Ethiopia; e Department of Radiology, Debre Tabor University, Debre Tabor, Ethiopia

**Keywords:** Sporadic lymphangioleiomyomatosis, LAM, Case report, Ethiopia

## Abstract

**Introduction and importance:**

Lymphangioleiomyomatosis (LAM) is a rare disorder of unknown cause which mostly affects young females and involving multi organ system with primarily involving lung.

**Presentation of case:**

A 35 year's old female Ethiopian known hypertension patient from Debre Tabor, Ethiopia, Africa; presented with progressively increasing cough with blood tingled sputum of 1–2 Arabic coffee cup per day, progressively increasing exertional shortness of breath and easy fatigability seven years back. Hypertensive and desaturate to level of 88 % at atmospheric air. There was diffuse crackle over posterior 2/3 chest with decrease air entry over lower 1/3 chest bilaterally. There is obstructive airway pattern on Spirometry. Multiple bilateral diffuse uniformly distributed cystic lesions on chest CT scan and angiomyolipoma in the kidneys on abdominal ultrasound. She is on supportive treatment, Budesonide/ Formoterol and amlodipine for the diagnosis of sporadic LAM.

**Clinical discussion:**

Sporadic LAM is a rare autosomal dominant disease affecting an estimated 3.4–7.8 per million individuals. While the pathophysiology remains unclear, LAM is thought to involve abnormal proliferation of smooth muscle-like cells, leading to cystic structures in axial lymphatics, lung cysts, and renal angiomyolipomas. Diagnosis is based on European Respiratory Society criteria, and management ranges from supportive care to definitive treatment depending on the patient's condition.

**Conclusion:**

Sporadic LAM is a disorder affecting multiple organ systems, primarily the lungs, and presents with diverse clinical manifestations. Even though it is difficult to diagnosis without lung biopsy; sporadic LAM can be diagnosed with imaging with compatible history in resource limiting setup.

## Highlights

• Rare case in around 3.4–7.8 per millions of individuals
• Diagnostic and treatment challenges in resource limiting setups.
• Sporadic LAM with different cystic organs(kidney, uterus and liver) involvement.

## 1 Introduction

Lymphangioleiomyomatosis is a rare, multi-systemic disorder that predominantly affects the lungs of young women. The underlying cause of LAM remains unknown [1,2]. Patients with LAM can be classified as either LAM-TSC or sporadic LAM. LAM-TSC refers to individuals who have both LAM and tuberous sclerosis complex (TSC). Sporadic LAM, on the other hand, describes LAM occurring without TSC [3]. LAM should be considered in young women presenting with a range of pulmonary symptoms such as unexplained dyspnea, spontaneous pneumothorax, cough, and hemoptysis [4]. Following a suggestive history, appropriate investigations, including tissue biopsy and/or high-resolution computed tomography (HRCT) scanning, are crucial for confirming a diagnosis of LAM [5]. Here, we present the case of a 35-year-old female diagnosed with sporadic LAM and essential hypertension, focusing on the diagnostic and management challenges encountered in a resource-limited setting, and highlighting the importance of considering these factors in clinical practice. This case report has been prepared following the SCARE (Surgical CAse REport) guidelines 2025 [6].

## 2 Case presentation

A 35-year-old female patient from Debre Tabor, Ethiopia, Africa, initially diagnosed with essential hypertension seven years prior, presented with a five-year history of progressively worsening symptoms on January 2023. These symptoms included a cough producing 1–2 Arabic coffee cups of blood‑tinged sputum per day, increasing shortness of breath with exertion, and easy fatigability.

Physical examination revealed hypertension (150–160/90–100 mmHg after treatment), a slightly elevated pulse rate (90–95 beats per minute) and respiratory rate (20–25 breaths per minute), a normal temperature (36.6 °C), and slightly low oxygen saturation (89–92 %) on room air. Chest examination revealed symmetrical chest movement with respiration, diffuse crackles over the posterior two-thirds of the chest, and decreased air entry over the lower third of the chest bilaterally.

Investigations revealed normal complete blood count and organ function tests. Abdominal ultrasound showed bilateral multiple small cystic lesions suggestive of angiomyolipoma in the kidneys and a 10 × 9 cm hypoechoic mass in the uterus. Echocardiography was normal. Spirometry demonstrated fixed airway obstruction (FEV1/FVC = 72–76 %.

**Fig. 1:**
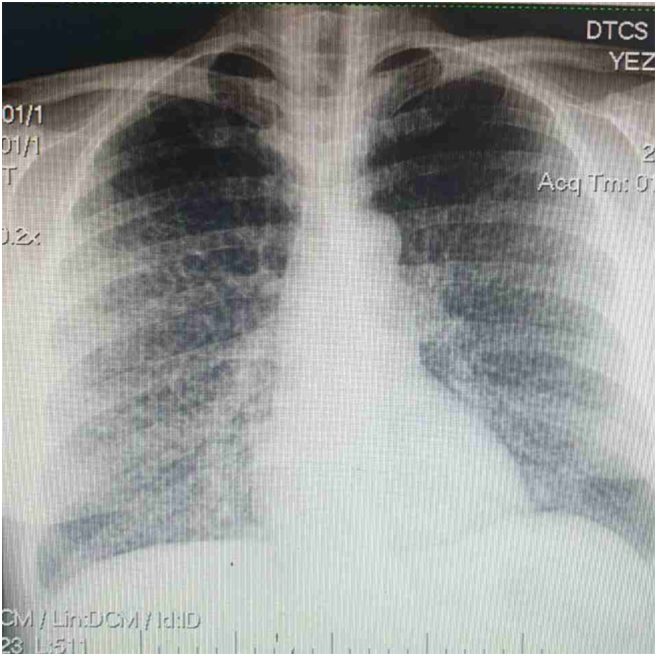
Bilateral diffuse reticular opacities.

Chest X-ray, as shown in Fig. 1,

This patient's HRCT imaging revealed findings consistent with sporadic LAM, a diagnosis further supported by the presence of a kidney angiomyolipoma, typical age, and female sex. While lung histopathology remains the diagnostic gold standard, it was not feasible in our setting. Given the high prevalence of infectious lung diseases in our patient population, we meticulously ruled out these alternative etiologies before applying the European Respiratory Society criteria, which the patient met, leading to a diagnosis of sporadic LAM. We then thoroughly discussed the diagnosis and treatment options with the patient.

The patient's treatment regimen began with Hydrochlorothiazide 25 mg daily, which was later switched to Amlodipine 10 mg daily for hypertension management. She was also prescribed salbutamol inhaler as needed and Beclomethasone 2 puffs twice daily for respiratory symptoms, alongside supportive measures such as chest physiotherapy, exercise, and trigger avoidance. Currently, the patient is on Symbicort (Budesonide/formoterol) and Amlodipine 10 mg daily. While her blood pressure is now well-controlled, she continues to experience fatigability, shortness of breath, and intermittent productive cough.

## 3 Discussion

This patient presents a classic case of LAM, demonstrating the typical demographic features of a young female with pulmonary manifestations and multi-organ involvement, including the kidneys and uterus. Sporadic LAM, a rare autosomal dominant disease, affects approximately 3.4–7.8 per million individuals, though the true prevalence remains largely unknown [2]. LAM is often misdiagnosed as asthma, emphysema, or COPD, highlighting the need for careful evaluation and consideration of this rare disorder in patients with similar symptoms [4].

While the exact pathophysiology of LAM remains unclear, it is believed to involve abnormal proliferation of smooth muscle-like cells. This leads to the formation of fluid-filled cystic structures within the axial lymphatics, the development of lung cysts, and renal angiomyolipomas. Mutations in the TSC1 or TSC2 genes, which code for hamartin and tuberin respectively, are thought to be involved. These genes play a role in regulating the mammalian target of rapamycin (mTOR) signaling pathway [7]. As our patient presented during her reproductive years, it is theorized that female sex hormones may play a role in the pathogenesis of LAM. This is supported by observations of exacerbations during periods of heightened hormone levels, such as pregnancy, menstruation, and hormonal contraception. However, the disease often stabilizes after menopause when hormone levels naturally decline [8].

Our patient presented with dyspnea and cough with blood‑tinged sputum, but did not exhibit pneumothorax or pleural effusion. Among the most common respiratory manifestations of LAM, shortness of breath is the most prevalent, affecting over 70 % of patients. Recurrent and bilateral primary pneumothorax occurs in 57 % of cases, while hemoptysis and pleural effusions are seen in 32 % and 12 % of cases, respectively [9,10]. Other extra pulmonary manifestations of LAM include lymphangioleiomyoma (29 %), renal angiolipomas (32 %), and less frequently renal hemorrhage, chylous effusion, and ascites [10]. Our patient exhibited renal angiomyolipoma, a common extrapulmonary manifestation of LAM, but did not present with other extrapulmonary features. While there's a well-established link between pulmonary hypertension and sporadic LAM, systemic hypertension is less commonly associated with sporadic LAM [10]. In this patient's case, the systemic hypertension is unlikely to be related to LAM because it preceded the respiratory symptoms and no LAM-related abnormalities were detected during the initial hypertension evaluation. While our patient demonstrated obstructive airway disease on pulmonary function testing, a DLCO was not performed. Airflow obstruction and a decreased diffusing capacity of the lung for carbon monoxide (DLCO) are the most common findings on pulmonary function tests in LAM, though DLCO can be normal in up to 30 % of patients [10]. Disease severity in LAM can be assessed using the forced expiratory volume in 1 s (FEV1), which was affected in our patient. The DLCO can also be used to assess disease severity, and its value often correlates with histological scores, CT findings, and exercise testing results [11,12]. While sporadic LAM is a rare disease, a few case reports have documented diverse presentations. For example, a case report by Rhee J et al. from the USA described a 55-year-old female patient who presented with pneumothorax following the onset of pleuritic chest pain [13].Similarly, a case report from a different institution in the USA by Kania B et al. documented a 39-year-old female presenting with chest pain, who was later found to have emphysema and pneumothorax, ultimately leading to a diagnosis of LAM [14]. A case report from Iran by Yousef Nikmanesh et al. describes a 31-year-old female who was diagnosed with LAM after presenting with shortness of breath for several weeks [15]. A study by Jaini M. Shah et al. analyzing 35 cases of LAM, with an average age of 38 (±14.41) and a predominance of females (78.78 %), revealed that the most common presentations were shortness of breath (60.6 %), pneumothorax (57.57 %), and chest pain (42.42 %). Extra pulmonary involvement was observed in 30.30 % of patients. Imaging studies, specifically CT scans, showed cystic lung changes in 81.25 % of patients, and decreased FEV1 was noted in 66.67 %. Our patient aligns with these common findings, being a female of a similar age, exhibiting decreased FEV1, and demonstrating cystic lung changes on CT scan [16]. To the best of our knowledge, there are no reported cases of LAM in Ethiopia.

**Fig. 2:**
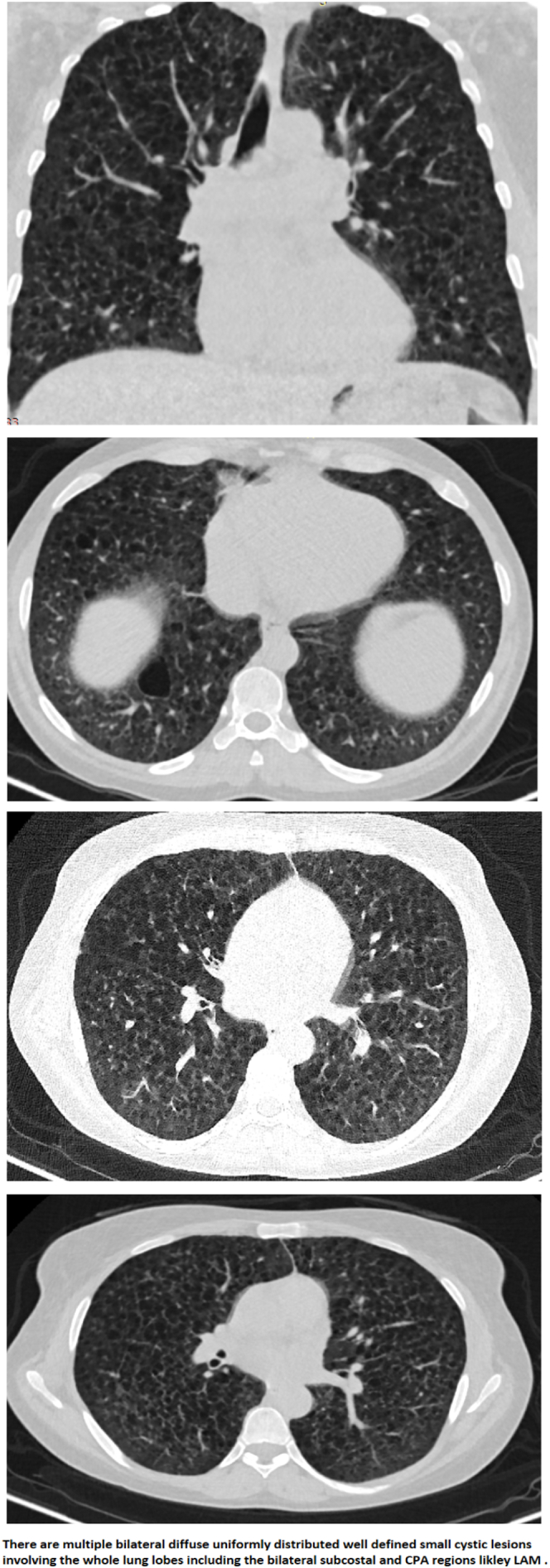
There are multiple bilateral diffuse uniformly distributed well defined small cystic lesions involving the whole lung bilaterally with no volume loss.

The patient's chest CT scan (shown in Fig. 2) revealed multiple, bilateral, diffuse, uniformly distributed, well-defined, small cystic lesions involving the entire lung bilaterally. Importantly, the lung volume appeared normal. These findings are consistent with the characteristic CT findings of LAM according to the European Respiratory Society guidelines [5]. Given these imaging findings, a lung biopsy would typically be the next step in confirming the diagnosis of LAM. However, as lung biopsy was not feasible for our patient, the characteristic pathological findings of LAM, which include multiple cysts with a multifocal nodular proliferation of immature perivascular epithelioid cells (LAM cells) and smooth muscle, should be considered in the diagnosis [17,18].

The diagnostic criteria for LAM diagnosis according to European respiratory society is [5];

### 3.1 Definite LAM

1) Characteristic or compatible lung High resolution computed tomography (HRCT), and lung biopsy fitting the pathological criteria for LAM; or.
2) Characteristic lung HRCT and any of the following: angiomyolipoma (kidney); thoracic or abdominal chylous effusion; lymphangioleiomyoma or lymph-node involved by LAM; and definite or probable TSC.

### 3.2 Probable LAM

1) Characteristic HRCT and compatible clinical history; or.
2) Compatible HRCT and any of the following: angiomyolipoma (kidney); and thoracic or abdominal chylous effusionc.

### 3.3 Possible LAM = Characteristic or compatible HRCT

#### 3.3.1 Remarks

1. HRCT features characteristic of LAM are multiple (>10) thin-walled round well-defined air-filled cysts with preserved or increased lung volume with no other significant pulmonary involvement specifically no interstitial lung disease with the exception of possible features of multifocal micronodular pneumocyte hyperplasia in patients with TSC.
2. HRCT features are compatible with pulmonary LAM when only few (>2 and < 10) cysts as described are present.
3. Compatible clinical features include pneumothorax (especially multiple and/or bilateral) and/or altered lung function tests as in LAM.

The management of patients with LAM emphasizes supportive measures such as avoiding cigarette smoking, encouraging the use of barrier methods, intrauterine devices (IUDs), or progesterone-only containing implants for contraception, and avoiding estrogen-based contraceptives [8,19]; Our patient does not smoke cigarettes and utilizes an IUD for contraception. She has not yet had children, which is recommended for patients with LAM due to the poor outcomes and exacerbations that can occur during pregnancy [20]. The pharmacological treatment of LAM primarily involves the use of mTOR inhibitors, specifically sirolimus or everolimus [21]; Due to the high cost and limited availability of mTOR inhibitors in Ethiopia, these medications were not prescribed for our patient. Bronchodilators, such as Budesonide/formoterol, are commonly used to manage respiratory symptoms. While our patient did not experience complications like pneumothorax or effusion, managing potential complications as they arise is also an important aspect of treatment [22]. For patients who experience progressive deterioration despite supportive and pharmacologic treatment, lung transplantation may be considered as a last resort [23].

## 4 Conclusion

Sporadic LAM is a rare disorder of unknown cause that predominantly affects young women, involving multiple organ systems, particularly the lungs. It can manifest as sporadic LAM or in conjunction with TSC as LAM-TSC. While definitive diagnosis often requires lung biopsy, a combination of characteristic imaging findings and compatible patient history can help establish a diagnosis.While pharmacologic treatments are available, they can be costly and limited in availability. Despite the diagnostic challenges of sporadic LAM, it should be considered in patients presenting with compatible HRCT findings, particularly in resource-limited settings like Ethiopia.

### 4.1 Strength and weakness

#### 4.1.1 Strengths

This case report details the clinical presentation and diagnostic process for a rare case of LAM in a resource-limited setting, where such reports are uncommon. It highlights unique diagnostic and management challenges specific to these settings.

#### 4.1.2 Weakness and limitations

The diagnosis was not confirmed with lung biopsy, and genetic studies were not performed due to resource limitations.

## Abbreviations

Cm: centimeter
CT: Computed tomography
DLCO: Diffusing capacity of the lung for carbon monoxide
Hgb: Hemoglobin
HRCT: High resolution computed tomography
LAM: Lymphangioleiomyomatosis
TSC: Tuberous Sclerosis Complex
WBC: White Blood Cell

## Consent

Written informed consent was obtained from the patient for publication and any accompanying images. A copy of the written consent is available for review by the Editor-in-Chief of this journal on request.

## Ethical approval

Ethical approval for this study was provided by our institution ethical review committee.

## Declaration of Generative AI and AI-assisted technologies in the writing process

AI language modelling tools were utilized for the improvement of English-language only in this case report.

## Funding

There is no source of funding found for this paper.

## Author contribution

AAA: Conceptualization, design of the study, acquisition of data, drafting the article, revising it critically for important intellectual content, approval of the version to be submitted.

DDM: Analysis, interpretation of data, drafting the article, revising it critically for important intellectual content, approval of the version to be submitted.

AMG: Conceptualization, analysis, drafting the article, revising it critically for important intellectual content, approval of the version to be submitted.

BDK: Acquisition of data, analysis, revising it critically for important intellectual content, approval of the version to be submitted.

FB: Acquisition of data, analysis, revising it critically for important intellectual content, approval of the version to be submitted.

AS: Acquisition of data, analysis, revising it critically for important intellectual content, approval of the version to be submitted.

## Guarantor

Addisu Assfaw Ayen, MD.

## Research registration number

N/A

## Conflict of interest statement

All authors declare that they have no conflict of interest.
